# Modulation of TRESK Background K^+^ Channel by Membrane Stretch

**DOI:** 10.1371/journal.pone.0064471

**Published:** 2013-05-15

**Authors:** Gerard Callejo, Jonathan P. Giblin, Xavier Gasull

**Affiliations:** 1 Neurophysiology Lab, Deptartment of Physiological Sciences I, Medical School, Universitat de Barcelona, Barcelona, Spain; 2 Institut d'Investigacions Biomèdiques August Pi i Sunyer (IDIBAPS), Barcelona, Spain; Universidad de Castilla-La Mancha, Spain

## Abstract

The two-pore domain K^+^ channel TRESK is expressed in dorsal root ganglion and trigeminal sensory neurons where it is a major contributor to background K^+^ current. TRESK acts as a break to prevent excessive sensory neuron activation and decreases in its expression or function have been involved in neuronal hyperexcitability after injury/inflammation, migraine or altered sensory perception (tingling, cooling and pungent burning sensations). All these effects have implicated this channel in nociception and mechanotransduction. To determine the role of TRESK in sensory transduction, we studied its sensitivity to changes in membrane tension (stretch) in heterologous systems, F-11 cells and trigeminal neurons. Laminar shear stress increased TRESK currents by 22–30%. An increase in membrane tension induced by cell swelling (hypotonic medium) produced a reversible elevation of TRESK currents (39.9%). In contrast, cell shrinkage (hypertonic solution) produced the opposite effect. Membrane crenators or cup-formers produced equivalent effects. In trigeminal sensory neurons, TRESK channels were mechanically stimulated by negative pressure, which led to a 1.51-fold increase in channel open probability. TRESK-like currents in trigeminal neurons were additively inhibited by arachidonic acid, acidic pH and hypertonic stimulation, conditions usually found after tissue inflammation. Our results show that TRESK is modulated by changes in cell membrane tension and/or cell volume. Several key players released during inflammation or tissue injury could modulate sensory neuron activation through small changes in membrane tension.

## Introduction

Background or leak potassium currents (K_2P_ family) have an important role in maintaining resting membrane potential in excitable and non-excitable cells. They display no voltage-dependence, which allow them to carry K^+^ currents over a wide range of membrane potentials. These properties make them key determinants of neuronal excitability, contributing to the likeliness of depolarizing stimuli to achieve action potential threshold, as well as shaping the neuron firing response (action potential duration and amplitude, repetitive firing, postdischarge) [1]–[4]. Among the several background K^+^ channels from the K_2P_ family expressed in dorsal root ganglion (DRG) and trigeminal (TG) sensory neurons [5]–[7], TREK-2 and TRESK currents account for 80% of the background current in small and medium-sized DRG neurons [8]. TRESK is highly expressed in sensory neurons and appears to play a significant role in setting up sensory neuron excitability under different pathological conditions: a significant down-regulation of TRESK was found in a neuropathic pain model [9] and changes in channel expression have been reported after inflammation [10]. In addition, a TRESK [G339R] functional knockout mice shows an enhanced DRG excitability [7] and a dominant-negative mutation in the human channel is linked to familial migraine with aura [11]. TRESK is also the target of sanshool, contained in Sichuan peppers, which produces numbing and tingling sensations [12]–[14]. Understanding more about the mechanisms by which TRESK activity is modulated will yield further insights into how the regulation of sensory neuron excitability is achieved.

Leak K^+^ channels, far from being passive players, are highly regulated by multiple physico-chemical factors including temperature, pH, hypoxia, volatile anesthetics and poly-unsaturated fatty acids. Furthermore, these channels can be modulated by PKA and/or PKC phosphorylation following stimulation of G_s_ or G_q_ coupled receptors [2], [4], [15]. Mechanical stimulation is another regulator of K_2P_ channel function: TREK-1, −2 and TRAAK are highly modulated by membrane stretch (possibly via their interaction with the actin cytoskeleton). Whether or not TRESK is also a stretch-sensitive channel is unknown to date. A study using radial stretch and hydroxy-alpha-sanshool described different populations of TRESK-expressing mechanosensitive sensory neurons, including stretch-sensitive large neurons expressing TRESK but not TRPV1 (likely low threshold mechanoreceptors or propioceptors) and stretch sensitive smaller neurons co-expressing TRESK and TRPV1 (likely non-peptidergic C-fiber nociceptors) [16]. In our previous study, we found that injection of the alkylamide synthetic derivative IBA (which blocks TRESK currents) into the rat hindpaw produced a decrease in the mechanical threshold to painful stimulation [9]. Similar effects were also observed upon knockdown of TRESK using siRNA [9]. These data suggest a role for TRESK in the modulation of mechanosensory responses.

In the present report, we have studied whether TRESK can be modulated by mechanical stimuli and if this stretch sensitivity can play a significant role in membrane currents of sensory neurons. Our findings show that TRESK currents are enhanced by shear stress, as well as by increasing membrane tension with a hypotonic stimulus. Manipulation of the membrane tension by exposure to membrane-deforming chemicals also modulates channel activity. Stretching the cell membrane by applying suction through a patch pipette also enhances channel opening at the single channel level. This newly described property of TRESK represents a further level of control in the fine-tuning of sensory neuron excitability. Its mechanosensitivity in addition to its modulation by different chemical stimuli will likely contribute to the control of sensory neuron excitability and suggests a possible role in touch detection and/or pain sensation.

## Materials and Methods

### Ethics statement

All experimental procedures involving animals were reviewed and approved by the University of Barcelona Animal Care Committee and by the Natural medium department of the Generalitat de Catalunya, Catalonia, Spain (Ref. 5853).

### Plasmid construction

Rat TRESK in the pcDNA3.1 vector (kindly provided by Dr. S. Yost, University of California-San Francisco) was subcloned into the pIRES_2_-EGFP (*NheI/EcoRI*) or pEGFP-C3 (*BamHI/XbaI*) vector and used for transient or stable transfection of cell lines as described below. Human TRESK pcDNA3.1 vector was kindly provided from Dr. Y. Sano (Astellas Pharma Inc, Ibaraki, Japan) and subcloned into pEGFP-C2 vector using *EcoRI/SmaI* digest. To construct the TRESK/TASK chimeras, the C-terminus of human TASK1 (amino acids 245–394) or human TASK3 (amino acids 245–374) was fused to the TM4 of rat TRESK (at amino acid 357) using the technique of splicing by overlap extension [17], [18]. Briefly, a PCR fragment consisting of the spliced TRESK/TASK cDNA was generated by a two-step PCR strategy and subcloned in-frame into pEGFP-C1-rTRESK by *EcoRV/EcoRI* digestion. Primer sequences and a more detailed strategy are available upon request. Human TASK1 and TASK3 cDNAs were kind gifts from Dr. R. Preisig-Müller (Philipps-Universität Marburg, Germany). C-terminus deletion mutants of rTRESK were generated from the pEGFP-rTRESK vector. Truncations of TRESK were constructed by generating PCR products containing premature stop codons at the appropriate amino acid positions (K379, Y389, C397). These PCR products were then subcloned into pEGFP-C1-rTRESK using *BglII/EcoRI* digest.

### HEK293 and F-11 cell line culture and transfection

HEK293T cells, cultured in DMEM with 10% FBS, 1% penicillin/streptomycin and 1% glutamine, were seeded in 12-mm dishes 24 h before transfection. Cells were transiently transfected with pIRES_2_-EGFP vector alone (control) or stably transfected with pIRES_2_-EGFP-rTRESK using FuGene transfection reagent (Roche), according to the manufacturer's instructions. pCD8-mTREK1 (kindly provided by Dr. F. Lesage, Institut de Pharmacologie Moléculaire et Cellulaire-CNRS, Valbonne, France) or pcDNA3.1-TASK3 were cotransfected with GFP. The F-11 cell line were kindly provided by Dr. E. Deval (Institut de Pharmacologie Moléculaire et Cellulaire-CNRS, Valbonne, France) and used as previously reported [19]–[21]. F-11 cells were cultured in Ham's F-12 medium (Sigma-Aldrich) supplemented with 15% FBS, 1x HAT (sodium hypoxanthine, aminopterin and thymidine), 200 mg/ml allo-4-hydroxy-L-proline (Sigma- Aldrich) and 1% antibiotics (penicillin/streptomycin, Sigma-Aldrich). One day after plating, cells were transiently transfected as described above. Cells were used for patch-clamp experiments 24–48 h after transfection. Both HEK293 and F-11 cells were cultured at 37°C and 5% CO_2_.

### Trigeminal ganglion neuron culture

Adult male Sprague-Dawley rats (Harlan; 100–150 g) were kept at 22°C with free access to food and water in an alternating 12 h light and dark cycle. Animals were sacrificed by decapitation under anesthesia (isoflurane) and both trigeminal ganglions (TG) were removed and maintained in cold (4–5°C) Ca^2+^ – and Mg^2+^ -free Phosphate Buffered Saline solution (PBS, Sigma) supplemented with 10 mM glucose, 10 mM Hepes, 100 U.I./mL penicillin, 100 µg/mL streptomycin until dissociation. Subsequently, ganglia were minced with iridectomy scissors and incubated with collagenase CLS I (1 mg/ml; Biochrome AG, Berlin) for 1 h 45 min followed by 15 min trypsin treatment (0.25%; Sigma) before mechanical dissociation with fire-polished Pasteur pipettes. The cell suspension was then layered on top of a 28/12.5% Percoll (Sigma) gradient to separate myelin and nerve debris from the neurons. Next, the cells were centrifuged at 1300 g for 10 min at room temperature. After removing the upper 4.5 ml of the Percoll gradient and adding 4 ml Dulbecco's Modified Eagle's Medium (DMEM) medium, cells were centrifuged at 1000 g for 6 min. Neurons found in the pellet were suspended in 2 ml DMEM +10% fetal bovine serum (FBS; Sigma), centrifuged at 1000 rpm for 5 min and re-suspended in the culture medium [DMEM +10% FBS, 100 µg/ml penicillin/streptomycin, 100 mg/mL L-glutamine]. Cell suspensions were transferred to poly-L-lysine/laminin-coated 12 mm-diameter glass coverslips and incubated at 37°C and 95% air, 5% CO_2_ up to 1 day before being used for patch-clamp electrophysiological recordings. No NGF or other growth factors were added.

### Electrophysiological recording

Electrophysiological recordings were performed with a patch-clamp amplifier (Axopatch 200B, Molecular Devices, Union City, CA). Patch electrodes were fabricated in a Flaming/Brown micropipette puller P-97 (Sutter instruments). Electrodes had a resistance between 2–4 MΩ when filled with intracellular solution (in mM): 140 KCl, 2.1 CaCl_2_, 2.5 MgCl_2_, 5 EGTA, 10 HEPES at pH 7.3. Bath solution (in mM): 145 NaCl, 5 KCl, 2 CaCl_2_, 2 MgCl_2_, 10 HEPES at pH 7.4. The osmolality of the isotonic solution was 310.6±1.8 mOsm/Kg. Hypotonic bath solution (−25%) was made by decreasing NaCl to 105 mM (235.6±5.4 mOsm/Kg). Sorbitol was added to isotonic bath solution to obtain +10% (342.1±2.3 mOsm/Kg) or +25% (385.3±4.2 mOsm/Kg) hypertonic solutions.

Membrane currents were recorded in the whole-cell patch clamp configuration, filtered at 2 kHz, digitized at 10 kHz and acquired with pClamp 9 software. Data was analyzed with Clampfit 9 (Molecular Devices) and Prism 4 (GraphPad Software, Inc., La Jolla, CA). Series resistance was always kept below 15 MΩ and compensated at 70–80%. All recordings were done at room temperature (22–23°C) except a group of experiments recording TRESK currents in HEK293 cells that were done at 32–33°C by heating up the recording chamber bath and the perfusion solution. Shear stress stimulation in transfected cell lines was achieved by increasing bath perfusion rate from 0 or 0.1 ml/min (baseline) to 1 ml/min (shear stress).

Recordings in dissociated neurons were restricted to small and medium TG neurons (<30 µm diameter; <45 pF), which largely correspond to nociceptive neurons. Recordings were done 24 h after dissociation at room temperature. For single-channel recordings in TG neuron membrane patches, care was taken to use gentle patches. Before the pipette entered the bath and until cell contact, positive pressure was applied. Slight suction of far less amplitude than the one applied to induce a detectable stretch activation of channels, was applied to form the gigaseal. Single channel currents were recorded following the procedure of Hamill et al. [22] in the cell-attached configuration of the patch clamp technique. In cell-attached recordings, the bath solution used was the same as described above. The patch pipette contained the intracellular solution described (high K^+^), thus almost symmetrical K^+^ concentrations should be achieved.

Single channel currents were sampled at 20 kHz, filtered at 3 kHz and stored on the hard disk system. Command potential was set at 0 mV (cell resting voltage) and depolarizing or hyperpolarizing pulses were applied. Holding potentials reported in this study were the original values indicated on the amplifier and were not corrected for the liquid junction potential. Currents flowing into the pipette were considered to be positive. For current-voltage relationship calculation, open channel amplitudes were calculated at each patch potential by use of all-point amplitude histograms or manually when few openings were seen (e.g., at high hyperpolarized voltages). Open-channel probability (NPo) was calculated as: NPo =  (A1+2A2+3A3+…+NAN)/(A0+ A1+A2+A3+…+AN) where A0 is the area under the curve of amplitude histograms corresponding to current in the closed state, and A1…AN represents the histograms area reflecting the different open-state current levels for 1 to N channels present in the patch. Histogram parameters were obtained from multiple least squares Gaussian fits of the data. The patch membrane was stretched by applying negative pressure (suction) to the back end of the patch pipette using a calibrated syringe. Suction was monitored with a pressure transducer (9162-0, Mallinckrodt, Northhampton, U.K.) that was calibrated using a water manometer. To compare effects of membrane stretch on open-channel probability, single channel recordings at +80 mV were obtained at atmospheric pressure (0 mmHg) and in the presence of suction (−30 mmHg) for a period of 30 s each. Data was analyzed to obtain an amplitude histogram from which NPo was calculated as described above.

### Drugs

Drugs were obtained from Sigma-Aldrich (Madrid, Spain) unless stated: Tetrodotoxin (TTX, 2 µM); 4-Aminopyridine (4-AP, 1 mM); Tetraethylammonium (TEA, 1 mM); Chloroform (CHCl_3_, 5 mM); Chlorpromazine (CPZ, 10 µM). Iberiotoxin (IbTx, 50 nM) was obtained from Calbiochem (Merck KGaA, Darmstadt, Germany). Isobutylalkenyl amide (IBA) was kindly provided by Givaudan commercial flavor stocks (Cincinnati, OH).

### Statistical analysis

Data are presented as mean±s.e.m. Statistical differences between different sets of data were assessed by performing paired (Wilcoxon matched pairs test) or unpaired (Mann-Whitney test) non-parametric tests.

## Results

### TRESK currents are modulated by shear stress and cell membrane tension

**Figure 1:**
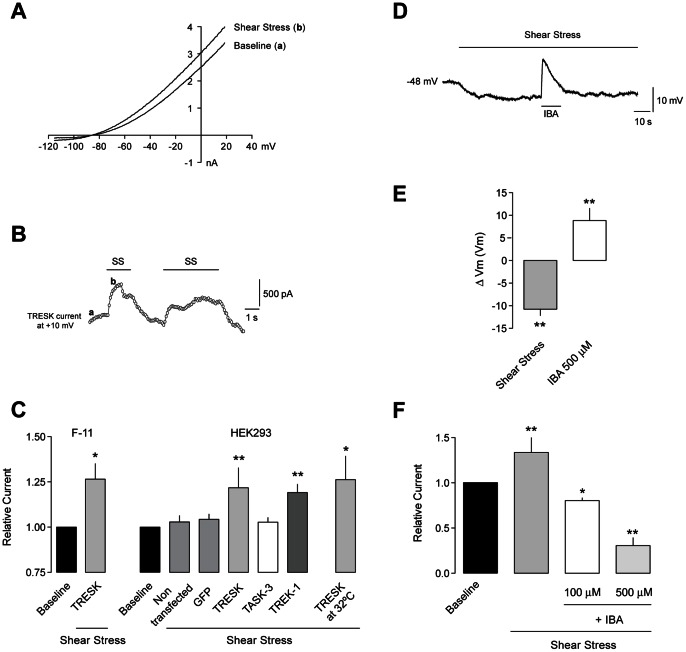
Shear stress modulates TRESK currents. **A.** An increase in the perfusion rate produces a potentiation of TRESK current in transfected F-11 cells. Representative current elicited by a voltage ramp is shown. **B.** Time course of the shear stress (SS) effect measured at +10 mV in a TRESK-transfected F-11 cell. Baseline current (a) is reversibly increased (b) when increasing perfusion rate. **C.** Quantification of the shear stress effects. TRESK-transfected F-11 cells (n = 6). HEK293-transfected cells: Non-transfected (n = 10), GFP (n = 9), TRESK (n = 8), TASK-3 (n = 5), TREK-1 (n = 9) and TRESK at 32°C (n = 4). **D.** Activation of TRESK channels by shear stress in HEK293 cells hyperpolarizes membrane potential. Addition of isobutylalkenyl amide (IBA, 500 µM) blocks TRESK and reverses the shear stress effect. **E.** Quantification of shear stress effect on membrane potential in HEK293 transfected cells (n = 7). **F.** Quantification of shear stress effect on whole-cell TRESK current in HEK293 transfected cells (n = 6) and block by IBA application (n = 6). *p<0.05; **p<0.01 Wilcoxon non-parametric test vs. baseline current/voltage.

Whole-cell TRESK currents were recorded in F-11 cells (a mouse neuroblastoma/rat DRG sensory neuron hybrid cell line) transfected with the pIRES2-EGFP-rTRESK expression vector. In F-11 cells expressing TRESK, a 1 s depolarizing ramp activated a prominent outward current with a strong outward-going rectification due to physiological solutions used (Fig. 1a) with a mean current at +10 mV of 4.73±0.83 nA (n = 6), while control non-transfected or eGFP-transfected cells showed much smaller endogenous currents (at +10 mV: 0.68±0.15 nA (n = 6) and 0.82±0.25 nA (n = 3), respectively). The reversal potential of the activated TRESK current was −73.3±0.6 mV (n = 6), which is close to the predicted value for *E*_K_^+^. When TRESK-expressing cells were challenged with an increase in bath perfusion rate (from 0 or 0.1 ml/min to 1 ml/min), a significant and consistent increase of TRESK current was observed (26.7±8.5% increase; p<0.05; Fig. 1a–c). This increase in current can be attributed to the increase in laminar shear stress experienced by the cells when the bath perfusion rate is increased. This effect was reversible and could be repeatedly induced without showing any sensitization (Fig. 1b). The increase in current magnitude (relative to the endogenous current) observed upon exposure to shear stress in F-11 cells over-expressing TRESK strongly suggests that it is TRESK mediating this increase. However, it cannot be formally ruled out that there are other contributions from other mechanosensitive channels potentially expressed in F-11 cells (TREK-1, for example). To confirm TRESK activation by shear stress, experiments were done using a stable HEK293 cell line expressing TRESK, where the same effect upon exposure to shear stress was observed (22.0±11% increase; p<0.01; Fig. 1c). In contrast, non-transfected or eGFP-transfected HEK cells only showed a small non-significant increase in current (2.9±3%, n = 10 and 4.3±3%, n = 9, respectively). Given that HEK cells are not likely to express other channels endogenously expressed in the neuronal hybrid F-11 cell line, this result strongly suggests that TRESK channels mediate this effect. In parallel, the human clone of the channel, transiently expressed in HEK293 cells, showed a similar activation upon shear stress stimulation (18–20% increase; data not shown). As a negative control, TASK-3, that has been reported to be mechanically insensitive [1], [23], did not show a significant activation in response to an increase in shear stress (2.8±2.5%; Fig. 1c). In contrast, TREK-1 current showed a significant increase to shear stress (19.2±4.6%; p<0.01; Fig. 1c) and to hypotonic shock (data not shown) as previously reported [1], which served as positive control. Although TRESK is not heat-sensitive itself [24], lipids surrounding the channel may be altered by temperature. Experiments performed at 32–33°C in transfected HEK cells showed that shear stress increased TRESK whole-cell current by 26.4±13.9% (p<0.05; Fig. 1c), confirming the results previously obtained at room temperature.

When measuring membrane voltage in current clamp mode, the shear stress effect produced a significant hyperpolarization of −10.8±1.4 mV (n = 7; p<0.01; Fig. 1d, e). Despite the lack of selective blockers for K_2P_ channels and in particular for TRESK, we have previously shown that the isobutylalkenyl amide (IBA) is a non-selective blocker of TRESK (Fig. 4 in [9]). Addition of this compound (IBA, 500 µM) reversed the hyperpolarization elicited by shear stress (Fig. 1d, e). The increase in TRESK whole-cell current due to shear stress could be also blocked by IBA application (Fig. 1f).

**Figure 4:**
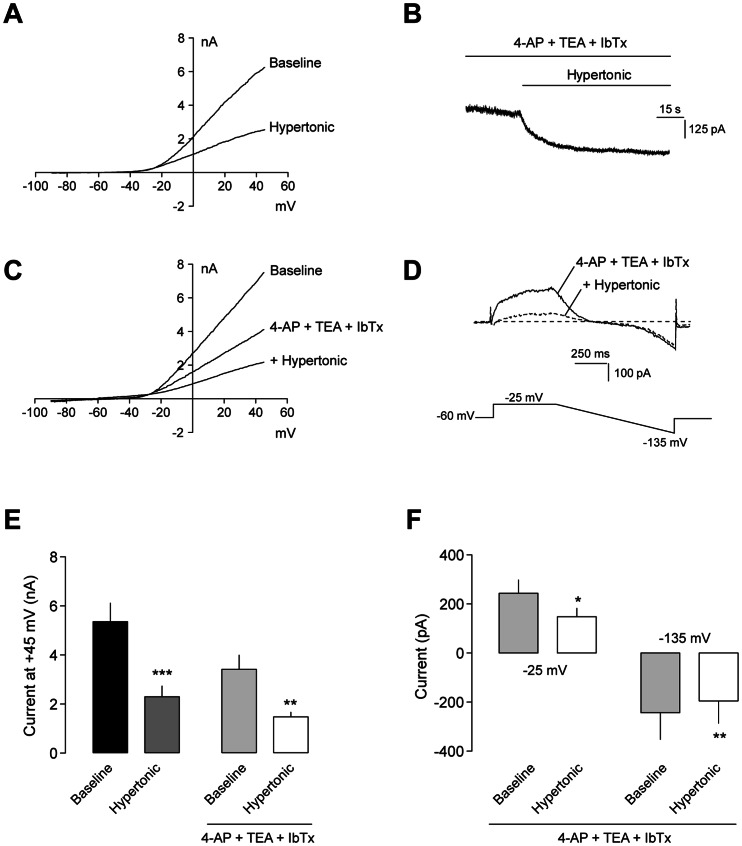
Membrane tension modulates TRESK-like currents in trigeminal sensory neurons. **A.** Total whole-cell K^+^ current of a small trigeminal sensory neuron in the presence of 2 µM TTX. Currents were elicited by a depolarizing ramp from −90 to +50 mV. Holding voltage was −60 mV. A 10% hypertonic solution produces a significant decrease of the current. **B.** Membrane current from a trigeminal neuron recorded at +20 mV in the presence of TTX (2 µM), 4-Aminopyridine (4-AP, 1 mM), Tetraethylammonium (TEA, 1 mM) and Iberiotoxin (IbTx, 50 nM). In these conditions and at room temperature, most of the current measured should be carried by TRESK. 10% hypertonic solution produces a significant decrease of the current. **C.** Similar experiment as in B but with total K^+^ current activated with a depolarizing ramp from −90 to +50 mV. **D.** Example of a recording in a trigeminal neuron using a protocol to minimize activation of voltage-gated transient K^+^ outward currents and in the presence of Na^+^ and K^+^ blockers. Hypertonic medium produced a significant reduction of the activated current. **E.** Quantification of the experiments performed in A (n = 11) and C (n = 8). **F.** Quantification of currents recorded as in D. Measurements were done at the end of the pulse (−25 mV) and at the end of the ramp (−135 mV; n = 8). *p<0.05; **p<0.01; ***p<0.001 Wilcoxon non-parametric test vs. baseline current.

**Figure 2:**
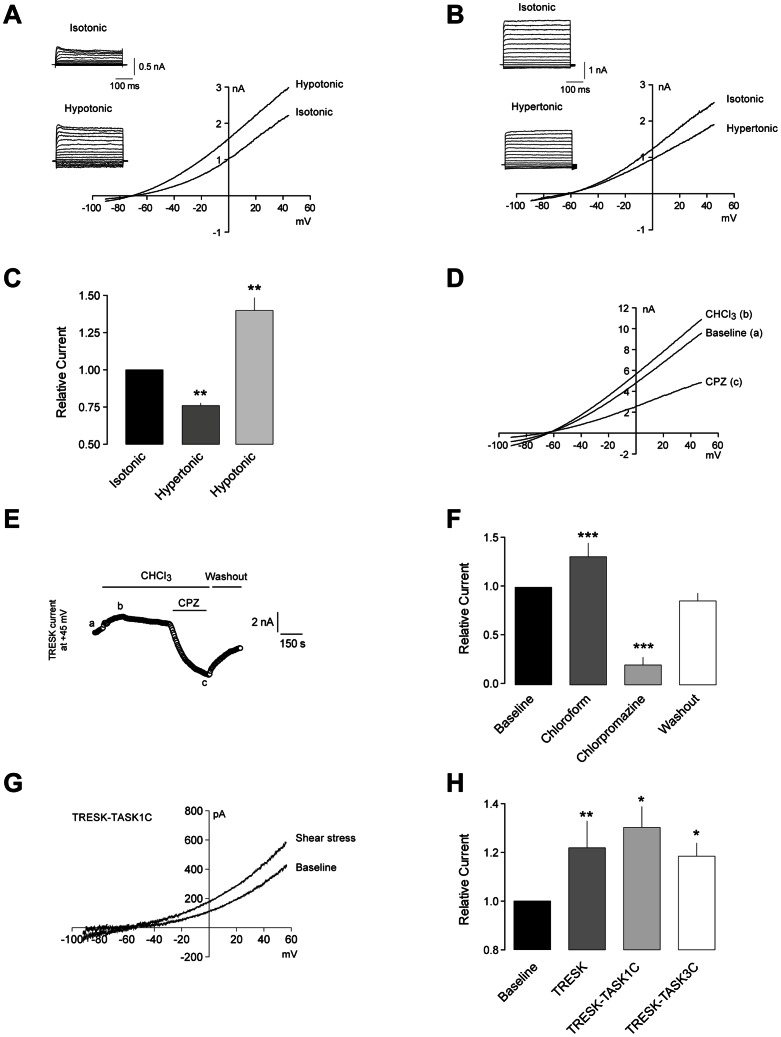
TRESK is regulated by changes in membrane tension. **A.** Membrane tension increase elicited by cell swelling in hypotonic medium produces a significant increase in TRESK currents in transfected HEK293 cell. Currents elicited by a voltage ramp from −90 to +50 mV in the whole-cell configuration. Holding voltage −60 mV. Inset: Currents elicited by a family of depolarizing voltage pulses in the same cell in isotonic and hypotonic conditions. **B.** Cell shrinkage in a hypertonic solution decreases membrane tension and reduces TRESK current. Inset: Currents elicited by a family of depolarizing voltage pulses in the same cell in isotonic and hypertonic conditions. **C.** Quantification of TRESK current modulation by changes in membrane tension in hypertonic and hypotonic media (n = 8 and 7, respectively). **D.** TRESK channel function is modulated by membrane curvature. Chloroform (b; 5 mM) and chlorpromazine (c; 10 µM) exert opposite effects on basal current (a). **E.** Time course of TRESK current measured at +45 mV from consecutive depolarizing voltage ramps (0.2 Hz). Basal current (a); chloroform (CHCl_3_; b); chlorpromazine (CPZ; c). **F.** Quantification of membrane curvature modulators on whole cell current in HEK293 cells expressing TRESK (n = 10). **G.** Effect of shear stress in TRESK-TASK1C chimeric channel expressed in HEK293 cells. **H.** Quantification of shear stress effect on TRESK (n = 8, reproduced from Fig. 1c for comparative purposes), TRESK-TASK1C (n = 4) and TRESK-TASK3C (n = 5) in HEK293 cells. *p<0.05; **p<0.01; ***p<0.001 Wilcoxon non-parametric test vs. baseline current.

To further study whether TRESK is modulated by mechanical stimuli, we used hypo- and hypertonic solutions to induce changes in membrane tension due to cell swelling or shrinkage, respectively. In TRESK-expressing HEK cells whole-cell currents recorded in hypotonic medium were significantly larger (39.9±22% n = 7, p<0.01) than those recorded in isotonic conditions both when recorded with a voltage ramp (Fig. 2a, c) or with voltage pulses (inset Fig. 2a). In contrast, a decrease in membrane tension produced the opposite effect: in hypertonic conditions a significant decrease in TRESK current was observed (-23.6±4.9%, n = 8, p<0.01; Fig. 2b, c). The results obtained favor the hypothesis that the channel activity can be modulated by changes in membrane tension. To further test this hypothesis, we challenged TRESK-expressing cells with agents which induce membrane deformation/curvature; a membrane crenator (Chloroform; 5 mM) and a cup-former (chlorpromazine; 10 µM), as previously described for other channels of the family [1], [25], [26]. Chloroform application produced a significant increase in TRESK current in all cells tested (31.5±14%, n = 10, p<0.001). In contrast, chlorpromazine application produced a large current inhibition in all the cells tested, which reached levels even below the baseline current (−79.8±8%, n = 10, p<0.001; Fig. 2d–f). Because K^+^ background channels are thought to have a prominent role at voltages close to the cell resting membrane potential, in the same experimental group we measured changes in holding current before and during exposure to chloroform and chlorpromazine. In TRESK-transfected HEK cells, baseline holding current was 489±127 pA (n = 10, Vhold  = −60 mV) showing that channels are active at rest. Chloroform application produced a positive shift in holding current (181±55%; p<0.001 vs. baseline), while chlorpromazine produced the opposite effect shifting the holding current to negative values (−346±94%; p<0.001 vs. baseline). This result indicates that membrane tension modifies channel activity in the whole voltage range, including membrane potentials close to the cell resting membrane voltage.

Previous studies on TREK-1 have shown that the C-terminal region of the channel interacts with membrane phospholipids through a cluster of charged amino acids and this C-terminal region is important for mechano-gating [1], [27], [28]. In an attempt to study whether TRESK stretch-activation also depends on the C-terminal domain, we produced truncated forms of the channel in the C-terminal region. Complete (ΔK379) or partial (ΔC397 or ΔY389) deletion of the C-terminal domain did not produce functional channels when transfected in HEK293 cells despite the fact that some GFP fluorescence could be seen in intracellular compartments of the cell. These results suggest that an intact C-terminal domain is critical for channel expression, trafficking and/or function. Similar results have been reported for TREK-1, TREK-2 and TRAAK where total or partial C-terminal deletions greatly reduced or totally abolished basal channel currents [1], [27], [29]. We next constructed chimeric channels where the C-terminus of TRESK had been replaced by that of TASK-1 or TASK-3, which are mechano-insensitive channels. When expressed in HEK293 cells, both chimeric channels display a reduced basal current compared to TRESK, but still were sensitive to laminar shear stress, which increased TRESK/TASK-1C current by 30.3±8% (n = 4, p<0.05; Fig. 2g, h) and TRESK/TASK-3C current by 18.5±5% (n = 5, p<0.05; Fig. 2 h). This is in agreement with a previous study, where replacement of the C-terminus of TRAAK channels with that of TASK1 or TASK3 did not affect the response of the channel to pressure [29].

In summary, the activity of TRESK channels expressed in heterologous systems or in a DRG cell line (F-11 cells) is modulated by changes in membrane tension and shear stress. This suggests that mechanical stimulation is a potentially novel regulator of channel activity.

### Stretch-activation of endogenous TRESK channels in trigeminal sensory neurons

**Figure 3:**
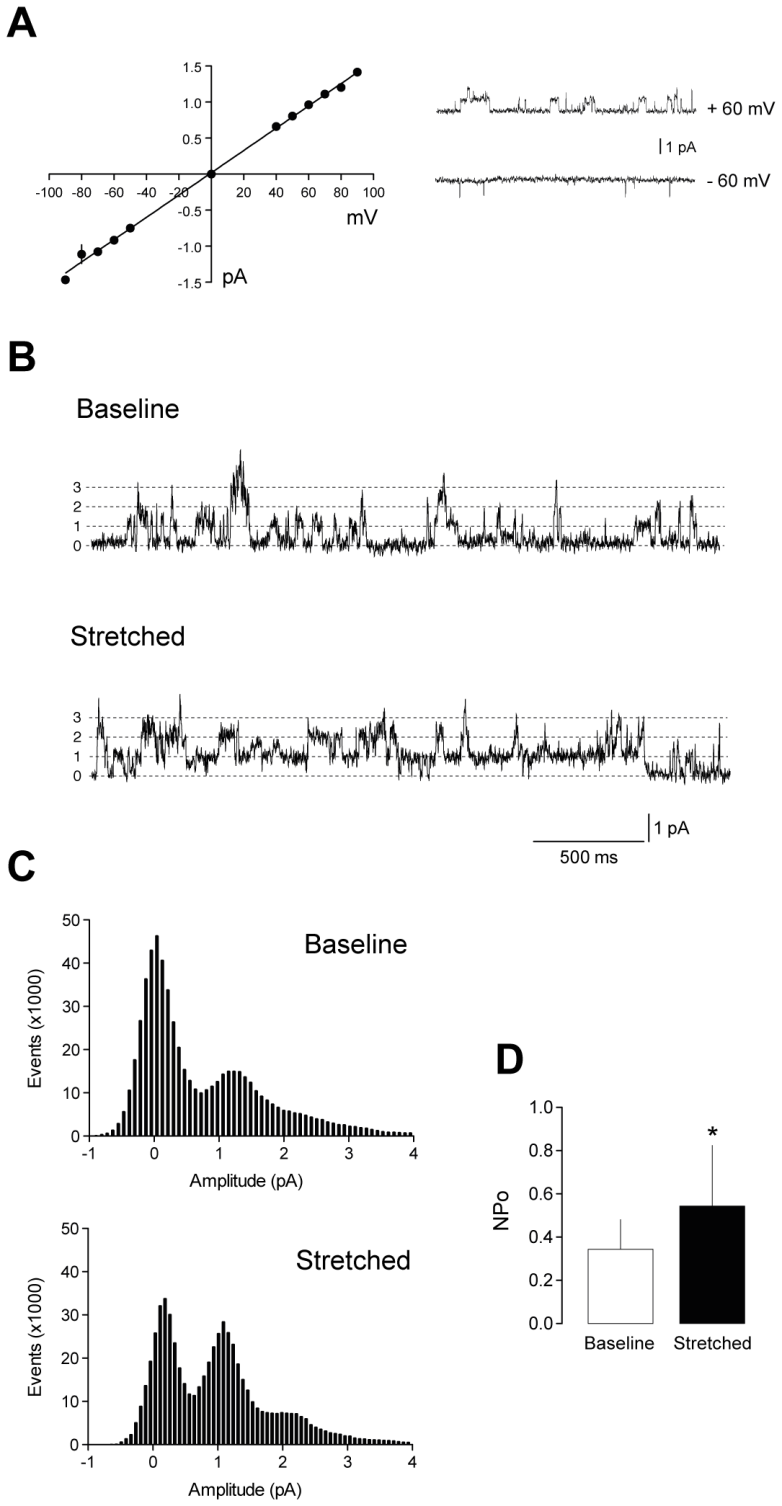
Membrane stretch modulates single channel TRESK currents identified in trigeminal sensory neurons. **A.** Current-voltage relationship of single channel openings at different membrane voltages (−90 to +90 mV) in cell-attached patches (n = 8). Holding voltage = 0 mV. High K^+^ solution was used in the recording pipette. Inset: examples of single channel currents obtained at +60 and −60 mV. **B.** TRESK single channel currents recorded at +80 mV in a cell attached patch of a small trigeminal neuron. Baseline recording was done at atmospheric pressure (0 mmHg). Mechanical stimulation (stretch) was performed by applying −30 mmHg suction through the patch pipette. **C.** Representative amplitude histograms in baseline and stretched conditions obtained from recordings performed as described in B. **D.** Quantification of NPo obtained from 6 cell-attached patches were TRESK was identified. *p<0.05 Wilcoxon non-parametric test vs. baseline NPo.

Next, we tested whether membrane stretch could modify TRESK channel activity in cell membrane patches of dissociated trigeminal sensory neurons. In small-sized trigeminal sensory neurons (likely nociceptors) where TRESK is expressed [13], this channel was identified by its single-channel properties and conductance at depolarized and hyperpolarized membrane potentials. In cell-attached patches (high K^+^ solution in the pipette), TRESK channels had a linear current-voltage relationship with a single-channel conductance of 15.4±0.2 pS (measured as the slope of the I-V relationship; n = 8; Fig. 3a), which is in the range of previously reported data [8], [30]. When identified TRESK channels in the cell-attached patches were mechanically stimulated by applying −30 mmHg suction through the patch pipette, an increase in channel activity was observed (Fig. 3b, c). Basal open probability at +80 mV was 0.34±0.13 (baseline, n = 6 cell-attached patches from 6 different neurons), similar to previously reported data [8]. Stretching the membrane produced a 1.51-increase in open probability to 0.54±0.28 (stretched, n = 6; p<0.05; Fig. 3d). Similar results were obtained when patches were held at negative voltages (data not shown), thus stretch activation appears to be independent of the voltage difference across the membrane.

### Modulation of TRESK-like currents by changes in membrane tension in trigeminal neurons

To study whether changes in membrane tension modulate TRESK currents in native conditions, we next challenged small trigeminal sensory neurons with a 10% hypertonic solution (342.1±2.3 mOsm/Kg) and recorded total K^+^ current. Hypertonic medium produced a 55.0±8.1% reduction of the total K^+^ current (n = 11; p<0.001; Fig. 4a), which is likely to include TRESK channels and other voltage-activated K^+^ conductances. Due to the lack of selective TRESK channel blockers/activators, we used a cocktail of drugs as well as recordings performed at room temperature in an attempt to minimize the contribution of other ionic conductances (and in particular other K^+^ channels) to the currents recorded. In addition to TTX (2 µM), the general potassium blockers 4-AP (1 mM) and TEA (1 mM) were used at concentrations known to block other K^+^ channels but not TRESK [8], [24], [31]. Iberiotoxin (50 nM) was also included to block Ca^2+^-dependent K^+^ channels. Other members of the K_2P_ family are likely to be present in small and medium-sized sensory neurons. According to other studies, TREK-2 and TRESK channels are major contributors to total background current, while TREK-1 and TRAAK carry a smaller fraction of the current [7], [8]. At room temperature, TREK-1, −2 and TRAAK are mostly inactivated, therefore under these conditions most of the background current should be carried by TRESK [8], [32]. In these experimental conditions, application of a hypertonic solution produced a 50.0±8.8% reduction of the holding current activated at +20 mV (Fig. 4b; n = 10; p<0.05).

When whole-cell current was activated by a voltage ramp (without drugs), the drug cocktail produced a significant decrease in total current (32.6±7.3%; n = 8; p<0.01; Fig. 4c), and a further reduction was achieved in hypertonic medium (49.7±7.9% vs. baseline with drugs; n = 8; p<0.01 Fig. 4c, e). As previously described [7], [9], a protocol to minimize activation of voltage-gated transient K^+^ outward currents was also used to measure the effect of hypertonic medium (Fig. 4d). Currents measured at the end of voltage pulse to −25 mV and ramp to −135 mV were significantly reduced by changes in membrane tension due to the hypertonic medium (Fig. 4d, f). In summary, changing the membrane tension by a mild hypertonic solution produces a significant decrease in TRESK current which is similar to that obtained when expressed in heterologous systems.

### Modulation of TRESK currents by inflammatory mediators

Previous data shows that TRESK channels are activated or inhibited by changes in membrane tension. Because TRESK is expressed in touch and nociceptive sensory neurons [7]–[10], [13], [16], its activation/inhibition could play a significant role in the detection of mechanical or other depolarizing stimuli. After tissue injury or inflammation, several potent inflammatory mediators are released in the interstitial fluid to form an exudate with an acidic and hyperosmotic content [33]–[35]. In contrast to other leak channels from the same family [1], [2], [8], [25], arachidonic acid (AA), a well-known inflammatory mediator, inhibits TRESK currents [24].

**Figure 5:**
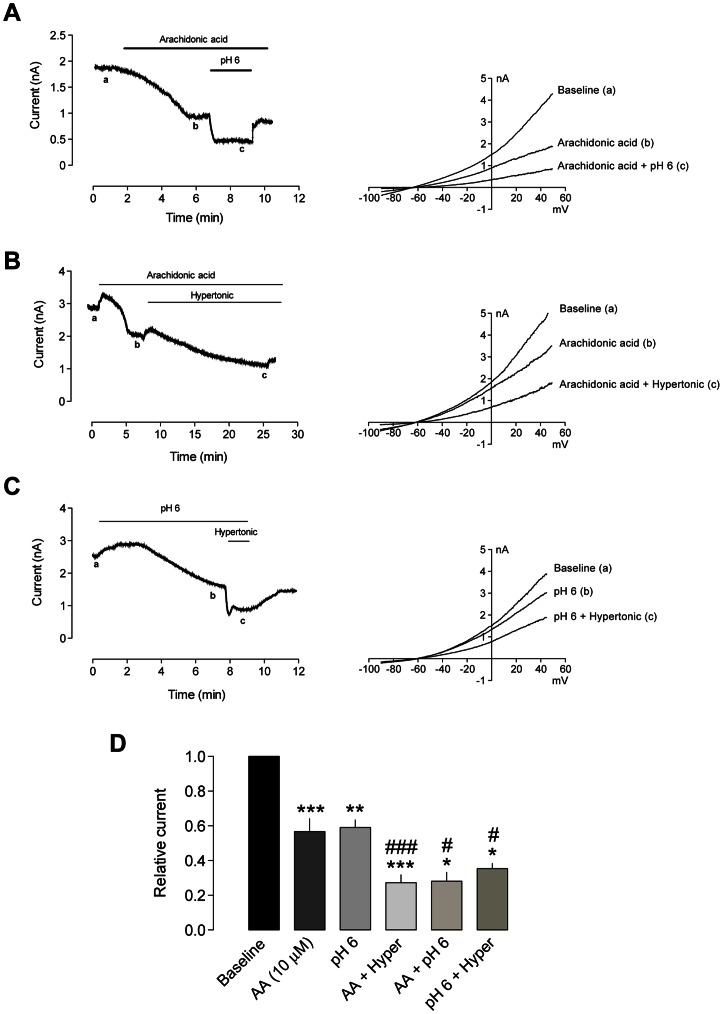
Additive inhibitory effects of inflammatory mediators and hyperosmotic conditions TRESK current. **A.** *Left:* TRESK current recorded at +20 mV in a transfected HEK293 cell. Additive inhibitory effects can be observed by application of arachidonic acid (AA, 10 µM) and acidic pH of 6. In a, b and c, a depolarizing ramp from −90 to +50 mV was recorded to obtain total K^+^ current (*right*). **B.** *Left:* AA and hypertonic conditions produce additive inhibitory effects on the TRESK current recorded at +20 mV in a transfected HEK293 cell. Notice that in some cases, a stimulatory shear stress effect can be seen when the bath perfusion is turned on. In a, b and c, depolarizing ramps from −90 to +50 mV were recorded to obtain the current-voltage relationship (*right*). **C.** *Left:* Acidic pH and hypertonic conditions have also additive effects on the TRESK current recorded at +20 mV in a transfected HEK293 cell. In a, b and c, depolarizing ramps from −90 to +50 mV were recorded to obtain the current-voltage relationship (*right*). D. Quantification of inhibitory effects of AA (n = 14), acidic pH (n = 6), AA + hypertonic medium (n = 10), AA + acidic pH (n = 6) and acidic pH + hypertonic medium (n = 6). *p<0.05; **p<0.01; ***p<0.001 Wilcoxon non-parametric test vs. baseline current. ^#^p<0.05; ^###^p<0.001 Wilcoxon non-parametric test vs. AA or pH 6.

To assess whether inflammatory mediators had additive effects with mechanical stimulation, we challenged HEK293 cells expressing TRESK channels with AA or acidic pH and then with hypertonic medium. As expected, AA (10 µM) produced a 42.6±4.2% decrease in TRESK current recorded at +20 mV (n = 14; p<0.001; Fig. 5a, d). In the presence of AA, a drop in pH (pH 7.4 to 6.0) produced a further decrease in TRESK current (71.9±5.2% vs. baseline current; p<0.05), showing that the effects were additive. Similarly, when TRESK-expressing cells were challenged with hypertonic medium in the presence of AA or in acidic pH, additive effects were also found. Arachidonic acid *plus* mechanical stimulation of TRESK currents due to the hypertonic medium produced a maximum current inhibition of 72.7±4.5% (n = 10; p<0.001; Fig. 5b, d), while acidic pH and hypertonic medium decreased TRESK current by 64.6±3.0% (n = 6; p<0.05; Fig. 5c, d). In summary, the additive effects of different inflammatory mediators, together with changes in membrane tension produces a significant reduction of TRESK currents. In sensory neurons, this will greatly facilitate neuronal activation in response to any depolarizing stimuli in conditions of tissue injury and/or inflammation.

## Discussion

Among the different K^+^ background channels expressed in TG and DRG sensory neurons, TRESK shows the highest level of expression [7], [9], [10] and carries, together with TREK-2, most of the K^+^ background current [7], [8]. In contrast to other K_2P_ channels expressed in sensory neurons, TRESK is activated by intracellular calcium increases through calcineurin, thus acting as a “brake” to prevent excessive activation of sensory neurons in response to depolarizing stimuli. Other channels like TREK-1 and −2 as well as TRAAK are highly regulated by temperature changes and appear to have a significant role in temperature perception and mediating neuroprotective effects [36], [37]. In contrast, TRESK is temperature-insensitive, down-regulated by injury and inhibited by inflammatory mediators like acidic pH or arachidonic acid [9], [24], which, in fact, highly activates other K_2Ps_ [1], [25]. Here we show that, in addition, TRESK is modulated by membrane stretch, a property likely to participate in the regulation of the channel function under different conditions.

Different types of mechano-activated channels have been described (for review see [26], [38]–[40]). Some of them appear to be mechanically gated like TRPA1 [41], [42] or the Piezo1 and Piezo2 proteins, recently shown to be involved in mechanotransduction in touch and nociceptive sensory neurons [43]–[45]. Other channels like the members of the DEG/ENaC family or TRPV4 channels seem to be modulated by mechanical stimuli or by changes in cell membrane tension (stretch) but it is unclear if they are directly activated by mechanical stimuli [38]. Among the K_2P_ family of K^+^ channels, TREK-1, TREK-2 and TRAAK have been shown to be sensitive to applied pressure, changes in osmolality and by modulators of the lipid bilayer in heterologous cells [1], [25], [46] while others like TASK-1 or TASK-3 remain mechano-insensitive. It has been proposed that TREK-1 mechanosensitivity arises from an as yet unknown interaction between the C-terminal domain and the plasma membrane. A region in the C-terminal domain (Val298 to Thr322) with charged amino acids was identified to be important but not sufficient for mechanical and chemical sensitivity (activation by arachidonic acid) since a chimera of a TASK channel with the TREK-1 C-terminus did not show mechanical/chemical activation [1], [28]. On the contrary, TRAAK, which also has an homologous C-terminal region, retains its mechano-sensitivity when its C-terminus is replaced by that of TASK-1 or TASK-3 [29]. Together these data suggest a different molecular sensor for TRAAK activation as also suggested from the recently described atomic structure of the channel [47]. Similarly to TREK-1 and TRAAK, we have found that TRESK is activated by cell swelling and inhibited by cell shrinkage in hypertonic medium, thus making it highly probable that changes in membrane curvature/shape/tension modify channel activity/opening. This is further demonstrated by the use of membrane crenators or cup-former substances, which preferentially insert into the external or internal leaflet of the bilayer, thereby changing membrane tension [48]. Other mechano-activated channels like TRPA1 have been also shown to be sensitive to changes in membrane curvature [49]. In addition, pressure or suction application through the patch pipette in the whole-cell configuration modifies TRESK current in a similar fashion (Gasull & Callejo unpublished observations). rTRESK channels have a much shorter C-terminus compared with TREKs or TRAAK. Despite the fact that it also has some charged amino acids that could interact with membrane phospholipids (**K**LMQN**R**LL**H**TY**K**TLMLFVCQ**RE**VSLPC), this region is dissimilar from the one identified in TREK and TRAAK. In an attempt to discern the role of the C-terminus in TRESK mechanosensitivity, we partially (ΔC397 or ΔY389) or totally (ΔK379) deleted this region but these constructs did not produce functional channels when expressed in heterologous systems. In contrast and similarly to that described for TRAAK, a chimera of TRESK containing the C-terminus of TASK-1 or TASK-3 was still activated by shear stress. Taken together, all these data favor the hypothesis that stretch sensitivity is not due to a direct interaction of the C-terminus with the membrane but rather a conformational change of the whole channel within the membrane bilayer, adopting conformational states with distinct functional properties in response to applied membrane tension, as proposed for other mechanosensitive channels [50]–[52]. Therefore, we propose that an increase in membrane tension and/or curvature serves to stabilize the conformational state of the channel towards a greater cross-sectional area, with the larger areas favored by increasing tension (for review see [50]). In this way, the action of several local or volatile anesthetics blocking or activating the channel could be mediated by their insertion in the membrane, as proposed for other channels [53]–[55].

Braun et al. have recently shown that TRESK is regulated through phosphorylation by MARK kinases in a cluster of serines (S274, 276 and 279) located in the intracellular loop of the channel [56]. MARK kinases are linked to regulation of neuronal polarity and/or microtubule cytoskeleton [57], [58], thus it is possible that TRESK has a significant role in these processes in sensory neurons. We have not tested whether the microtubule or the actin cytoskeleton has some influence in TRESK regulation by mechanical stimulation. As previously stated, the effects of membrane crenators/cup-formers, which are not likely to modify the cell cytoskeleton but still modulate TRESK currents, point to a modulation of the channel through direct changes in membrane tension rather than an effect mediated by the cytoskeleton. Data from other K_2P_ channels shows that mechanical activation does not require the integrity of the cytoskeleton and can be achieved in excised membrane patches or in the presence of the cytoskeleton disrupters colchicine and cytochalasin D [1], [25]. TRESK channels seem to follow a similar mechanism of activation by mechanical stimuli where the activating force comes from the lipid bilayer. Since TREK and TRAAK channels are activated by arachidonic acid, it has been suggested that the mechanically sensitive phospholipase A_2_ could mediate these effects [25] although TRAAK mechanical activation is still possible in the presence of phospholipase A_2_ inhibitors. In contrast, TRESK is inhibited by arachidonic acid, but the channel can still be modulated by mechanical stimuli. This suggests that, in the case of TRESK, the modulatory effects of arachidonic acid and stretch follow independent mechanisms.

It is well known that mechanical stimulation activates sensory neurons [52]. Radial stretch on cultured DRG neurons activates nociceptors and mechanoreceptors [16]. Using hydroxy-alpha-sanshool and different TRP activators, two populations of stretch-sensitive neurons were described: low threshold mechanoreceptors/proprioceptors and non-peptidergic C-fiber nociceptors. Both these populations of stretch-sensitive neurons appear to express TRESK channels [16]. We and others have previously shown that blocking or decreasing TRESK expression increases mechanical sensitivity in the rat [9], [59]. Others have shown similar effects in lingual tactile sensitivity after IBA administration [60] or hydroxy-alpha-sanshool [61]. Mechanical regulation of TRESK channels may play a role in modulating responses to light touch, as well as painful mechanical stimuli, as also proposed by others [13], [14], [62]. In fact, here we show that TRESK expressed in heterologous systems is inhibited by arachidonic acid, acidic pH and by a slightly hypertonic medium in an additive manner. This implies that mechanical and chemical modulation of the channel has additive effects. These conditions are commonly found locally after tissue injury and/or inflammation, but also after peripheral nerve injury, which produces an increase of spinal arachidonic acid content [63]. Therefore, it is possible that, in order to trigger sensory neuron activation and/or persistent neuronal hyperexcitability after tissue/nerve injury, TRESK currents are diminished to facilitate neuronal activation by depolarizing stimuli. In addition to a possible regulation at the protein level, we also found a decrease in TRESK mRNA expression after nerve injury [9], both effects favoring a decrease in TRESK function in sensory neurons. The reported effects of modulators of K_2P_ channels (hydroxy-alpha-sanshool, IBA) on sensory perception (tingling, burning) [60], [61] are likely to be due to activation of different types of sensory fibers when applied to the skin-nerve preparation [14], on lingual sensory fibers [61] or sciatic C-fibres [9]. In fact, these substances produce an activation of tactile- and cooling-sensitive fibers as well as nociceptors [14], [61] but also some silent fibers are activated by these compounds [9], [61]. Interestingly, after administration of K_2P_ modulators, the sensitivity of many fibers is altered. Some fibers are sensitized to tactile stimulation [9], [59]–[61] or they are then able to be excited by stimuli to which they had previously been insensitive [61]. These effects have been proposed to mediate the tingling sensations reported after consumption of fruits containing natural alkylamides [13], [60]. In a similar way to alkylamides, it is possible to hypothesize that stretch-mediated activation/inhibition of TRESK could modulate mechanosensory transduction in peripheral sensory fibers like the sensitization of mechano-insensitive fibers observed after repetitive stimulation [64]. It is known that inflammatory mediators can acutely sensitize mechano-insensitive “sleeping” nociceptors and render them mechano-responsive [64], [65]. At least part of these effects could be mediated by the inhibitory effects of arachidonic acid and acidic pH on TRESK and further potentiated by the stretch-sensitivity of TRESK in the hypertonic conditions encountered in inflamed tissues. In a similar way, stretching the membrane provides TRAAK with a greater sensitivity to pH and arachidonic acid [29] **.** It is possible that substances that activate TRESK like the volatile anesthetic isoflurane decreases mechanical sensitivity due to mechanoactivation of the channel by means of changes in membrane tension. Because TRESK can be activated or inhibited depending on how a mechanical stimulus is applied (e.g. negative vs. positive pressure to the membrane), mechanical stimulation could facilitate neuronal activation (TRESK inhibition) or prevent it (TRESK activation). This hypothesis remains to be tested in future studies.

A dominant-negative mutation in TRESK has been associated with familial migraine with aura [11]. A subsequent study demonstrated that another mutation that produces a complete loss of channel function has been found in migraine and control cohorts, indicating that a single non-functional TRESK variant is not alone sufficient to cause typical migraine [66]. Nevertheless, an increase in sensory neuron excitability has been reported in TRESK[G339R] functional knockout mice [7], implying that loss of TRESK function favors an enhanced excitability in sensory neurons and in particular, in nociceptors. In migraine, cortical spreading depression activates trigeminal nociceptors producing release of pro-inflammatory peptides that cause neurogenic inflammation and may lead to central sensitization [67]. Mutations producing loss or a diminished function of TRESK are likely to facilitate nociceptor activation and enhance the likeliness of having a migraine episode. It is possible to speculate that substances that modify membrane tension could decrease TRESK function and facilitate activation of nociceptors when cortical spreading depression occurs in migraneurs.

In summary, we show here that TRESK possess an intrinsic sensitivity to changes in membrane tension. During tissue injury or inflammation, stimuli such as small osmotic changes will be able to modulate the channel, in addition to the inhibitory effect of inflammatory mediators like arachidonic acid or acidic pH. The combination of all these factors will down-regulate TRESK currents to enhance sensory neuron excitability. It remains to be elucidated in future studies if this effect contributes to the generation of tingling and burning sensations as well as mechanical allodynia evoked when blocking TRESK or some other K_2P_ channels. In addition, TRESK stretch-sensitivity might play a significant role in cellular processes involving the cytoskeleton, especially since the recently described regulation of TRESK channels by MARK kinases and their relationship with neuronal polarity.

## References

[1] Patel AJ, Honoré E, Maingret F, Lesage F, Fink M, et al (1998) A mammalian two pore domain mechano-gated S-like K+ channel. EMBO J 17: 4283–4290 doi:10.1093/emboj/17.15.4283. 9687497 10.1093/emboj/17.15.4283 PMC1170762

[2] Enyedi P, Czirják G (2010) Molecular background of leak K+ currents: two-pore domain potassium channels. Physiological Reviews 90: 559–605 doi:10.1152/physrev.00029.2009. 20393194 10.1152/physrev.00029.2009

[3] Brickley SG, Aller MI, Sandu C, Veale EL, Alder FG, et al (2007) TASK-3 two-pore domain potassium channels enable sustained high-frequency firing in cerebellar granule neurons. Journal of Neuroscience 27: 9329–9340 doi:10.1523/JNEUROSCI.1427-07.2007. 17728447 10.1523/JNEUROSCI.1427-07.2007 PMC6673138

[4] Noël J, Sandoz G, Lesage F (2011) Molecular regulations governing TREK and TRAAK channel functions. Channels (Austin) 5: 402–409 doi:10.4161/chan.5.5.16469. 21829087 10.4161/chan.5.5.16469 PMC3265763

[5] Yamamoto Y, Hatakeyama T, Taniguchi K (2009) Immunohistochemical colocalization of TREK-1, TREK-2 and TRAAK with TRP channels in the trigeminal ganglion cells. Neuroscience Letters 454: 129–133 doi:10.1016/j.neulet.2009.02.069. 19429069 10.1016/j.neulet.2009.02.069

[6] Talley EM, Solorzano G, Lei Q, Kim D, Bayliss DA (2001) Cns distribution of members of the two-pore-domain (KCNK) potassium channel family. Journal of Neuroscience 21: 7491–7505. 11567039 10.1523/JNEUROSCI.21-19-07491.2001 PMC6762917

[7] Dobler T, Springauf A, Tovornik S, Weber M, Schmitt A, et al (2007) TRESK two-pore-domain K+ channels constitute a significant component of background potassium currents in murine dorsal root ganglion neurones. J Physiol (Lond) 585: 867–879 doi:10.1113/jphysiol.2007.145649. 17962323 10.1113/jphysiol.2007.145649 PMC2375503

[8] Kang D, Kim D (2006) TREK-2 (K2P10.1) and TRESK (K2P18.1) are major background K+ channels in dorsal root ganglion neurons. Am J Physiol, Cell Physiol 291: C138–C146 doi:10.1152/ajpcell.00629.2005. 16495368 10.1152/ajpcell.00629.2005

[9] Tulleuda A, Cokic B, Callejo G, Saiani B, Serra J, et al (2011) TRESK channel contribution to nociceptive sensory neurons excitability: modulation by nerve injury. Molecular pain 7: 30 doi:10.1186/1744-8069-7-30. 21527011 10.1186/1744-8069-7-30 PMC3095542

[10] Marsh B, Acosta C, Djouhri L, Lawson SN (2012) Leak K^+^ channel mRNAs in dorsal root ganglia: relation to inflammation and spontaneous pain behaviour. Molecular and cellular neurosciences 49: 375–386 doi:10.1016/j.mcn.2012.01.002. 22273507 10.1016/j.mcn.2012.01.002 PMC3334831

[11] Lafrenière RG, Cader MZ, Poulin J-F, Andres-Enguix I, Simoneau M, et al (2010) A dominant-negative mutation in the TRESK potassium channel is linked to familial migraine with aura. Nat Med 16: 1157–1160 doi:10.1038/nm.2216. 20871611 10.1038/nm.2216

[12] Tsunozaki M, Bautista DM (2009) Mammalian somatosensory mechanotransduction. Curr Opin Neurobiol 19: 362–369 doi:10.1016/j.conb.2009.07.008. 19683913 10.1016/j.conb.2009.07.008 PMC4044613

[13] Bautista DM, Sigal YM, Milstein AD, Garrison JL, Zorn JA, et al (2008) Pungent agents from Szechuan peppers excite sensory neurons by inhibiting two-pore potassium channels. Nat Neurosci 11: 772–779 doi:10.1038/nn.2143. 18568022 10.1038/nn.2143 PMC3072296

[14] Lennertz RC, Tsunozaki M, Bautista DM, Stucky CL (2010) Physiological Basis of Tingling Paresthesia Evokedby Hydroxy-alpha-Sanshool. Journal of Neuroscience 30: 4353–4361 doi:10.1523/JNEUROSCI.4666-09.2010. 20335471 10.1523/JNEUROSCI.4666-09.2010 PMC2852189

[15] Enyedi P, Braun G, Czirják G (2012) TRESK: the lone ranger of two-pore domain potassium channels. Mol Cell Endocrinol 353: 75–81 doi:10.1016/j.mce.2011.11.009. 22115960 10.1016/j.mce.2011.11.009

[16] Bhattacharya MRC, Bautista DM, Wu K, Haeberle H, Lumpkin EA, et al (2008) Radial stretch reveals distinct populations of mechanosensitive mammalian somatosensory neurons. Proceedings of the National Academy of Sciences 105: 20015–20020 doi:10.1073/pnas.0810801105. 10.1073/pnas.0810801105 PMC2604979 19060212

[17] Higuchi R, Krummel B, Saiki RK (1988) A general method of in vitro preparation and specific mutagenesis of DNA fragments: study of protein and DNA interactions. Nucleic Acids Res 16: 7351–7367. 3045756 10.1093/nar/16.15.7351 PMC338413

[18] Horton RM, Hunt HD, Ho SN, Pullen JK, Pease LR (1989) Engineering hybrid genes without the use of restriction enzymes: gene splicing by overlap extension. Gene 77: 61–68. 2744488 10.1016/0378-1119(89)90359-4

[19] Francel PC, Harris K, Smith M, Fishman MC, Dawson G, et al (1987) Neurochemical characteristics of a novel dorsal root ganglion X neuroblastoma hybrid cell line, F-11. J Neurochem 48: 1624–1631. 2435852 10.1111/j.1471-4159.1987.tb05711.x

[20] Deval E, Noël J, Lay N, Alloui A, Diochot S, et al (2008) ASIC3, a sensor of acidic and primary inflammatory pain. EMBO J 27: 3047–3055 doi:10.1038/emboj.2008.213. 18923424 10.1038/emboj.2008.213 PMC2585165

[21] Delaunay A, Gasull X, Salinas M, Noël J, Friend V, et al (2012) Human ASIC3 channel dynamically adapts its activity to sense the extracellular pH in both acidic and alkaline directions. Proceedings of the National Academy of Sciences 109: 13124–13129 doi:10.1073/pnas.1120350109. 10.1073/pnas.1120350109 PMC3420194 22829666

[22] Hamill OP, Marty A, Neher E, Sakmann B, Sigworth FJ (1981) Improved patch-clamp techniques for high-resolution current recording from cells and cell-free membrane patches. Pflugers Arch 391: 85–100. 6270629 10.1007/BF00656997

[23] Kim Y, Bang H, Kim D (2000) TASK-3, a new member of the tandem pore K(+) channel family. J Biol Chem 275: 9340–9347. 10734076 10.1074/jbc.275.13.9340

[24] Sano Y, Inamura K, Miyake A, Mochizuki S, Kitada C, et al (2003) A novel two-pore domain K+ channel, TRESK, is localized in the spinal cord. Journal of Biological Chemistry 278: 27406. 12754259 10.1074/jbc.M206810200

[25] Maingret F, Fosset M, Lesage F, Lazdunski M, Honoré E (1999) TRAAK is a mammalian neuronal mechano-gated K+ channel. J Biol Chem 274: 1381–1387. 9880510 10.1074/jbc.274.3.1381

[26] Folgering JHA, Sharif-Naeini R, Dedman A, Patel A, Delmas P, et al (2008) Molecular basis of the mammalian pressure-sensitive ion channels: focus on vascular mechanotransduction. Prog Biophys Mol Biol 97: 180–195 doi:10.1016/j.pbiomolbio.2008.02.006. 18343483 10.1016/j.pbiomolbio.2008.02.006

[27] Chemin J, Patel AJ, Duprat F, Lauritzen I, Lazdunski M, et al (2005) A phospholipid sensor controls mechanogating of the K+ channel TREK-1. EMBO J 24: 44–53 doi:10.1038/sj.emboj.7600494. 15577940 10.1038/sj.emboj.7600494 PMC544907

[28] Maingret F, Patel AJ, Lesage F, Lazdunski M, Honore E (1999) Mechano- or acid stimulation, two interactive modes of activation of the TREK-1 potassium channel. J Biol Chem 274: 26691–26696. 10480871 10.1074/jbc.274.38.26691

[29] Kim Y, Bang H, Gnatenco C, Kim D (2001) Synergistic interaction and the role of C-terminus in the activation of TRAAK K+ channels by pressure, free fatty acids and alkali. Pflugers Arch 442: 64–72 doi:10.1007/s004240000496. 11374070 10.1007/s004240000496

[30] Kang D, Kim G-T, Kim E-J, La J-H, Lee J-S, et al (2008) Lamotrigine inhibits TRESK regulated by G-protein coupled receptor agonists. Biochem Biophys Res Commun 367: 609–615 doi:10.1016/j.bbrc.2008.01.008. 18190784 10.1016/j.bbrc.2008.01.008

[31] Liu C, Au JD, Zou HL, Cotten JF, Yost CS (2004) Potent activation of the human tandem pore domain K channel TRESK with clinical concentrations of volatile anesthetics. Anesth Analg 99: 1715–1722, tableofcontents. doi:10.1213/01.ANE.0000136849.07384.44. 10.1213/01.ANE.0000136849.07384.44 15562060

[32] Kang D, Choe C, Kim D (2005) Thermosensitivity of the two-pore domain K+ channels TREK-2 and TRAAK. J Physiol (Lond) 564: 103. 15677687 10.1113/jphysiol.2004.081059 PMC1456046

[33] Vakili C, Ruiz-Ortiz F, Burke JF (1970) Chemical and osmolar changes of interstitial fluid in acute inflammatory states. Surg Forum 21: 227–228. 5514733

[34] Steen KH, Steen AE, Reeh PW (1995) A dominant role of acid pH in inflammatory excitation and sensitization of nociceptors in rat skin, in vitro. J Neurosci 15: 3982–3989. 7751959 10.1523/JNEUROSCI.15-05-03982.1995 PMC6578188

[35] Steen KH, Reeh PW, Anton F, Handwerker HO (1992) Protons selectively induce lasting excitation and sensitization to mechanical stimulation of nociceptors in rat skin, in vitro. J Neurosci 12: 86–95. 1309578 10.1523/JNEUROSCI.12-01-00086.1992 PMC6575698

[36] Heurteaux C, Guy N, Laigle C, Blondeau N, Duprat F, et al (2004) TREK-1, a K+ channel involved in neuroprotection and general anesthesia. EMBO J 23: 2684–2695 doi:10.1038/sj.emboj.7600234. 15175651 10.1038/sj.emboj.7600234 PMC449762

[37] Noël J, Zimmermann K, Busserolles J, Deval E, Alloui A, et al (2009) The mechano-activated K+ channels TRAAK and TREK-1 control both warm and cold perception. EMBO J 28: 1308–1318 doi:10.1038/emboj.2009.57. 19279663 10.1038/emboj.2009.57 PMC2683043

[38] Árnadóttir J, Chalfie M (2010) Eukaryotic mechanosensitive channels. Annu Rev Biophys 39: 111–137 doi:10.1146/annurev.biophys.37.032807.125836. 20192782 10.1146/annurev.biophys.37.032807.125836

[39] Bautista DM, Lumpkin EA (2011) Perspectives on: information and coding in mammalian sensory physiology: probing mammalian touch transduction. J Gen Physiol 138: 291–301 doi:10.1085/jgp.201110637. 21875978 10.1085/jgp.201110637 PMC3171080

[40] Nilius B, Honoré E (2012) Sensing pressure with ion channels. Trends Neurosci 35: 477–486 doi:10.1016/j.tins.2012.04.002. 22622029 10.1016/j.tins.2012.04.002

[41] Vilceanu D, Stucky CL (2010) TRPA1 mediates mechanical currents in the plasma membrane of mouse sensory neurons. PLoS ONE 5: e12177 doi:10.1371/journal.pone.0012177. 20808441 10.1371/journal.pone.0012177 PMC2922334

[42] Brierley SM, Castro J, Harrington AM, Hughes PA, Page AJ, et al.. (2011) TRPA1 contributes to specific mechanically activated currents and sensory neuron mechanical hypersensitivity. J Physiol (Lond). doi:10.1113/jphysiol.2011.206789. 10.1113/jphysiol.2011.206789 PMC3167119 21558163

[43] Coste B, Xiao B, Santos JS, Syeda R, Grandl J, et al (2012) Piezo proteins are pore-forming subunits of mechanically activated channels. Nature 483: 176–181 doi:10.1038/nature10812. 22343900 10.1038/nature10812 PMC3297710

[44] Coste B, Mathur J, Schmidt M, Earley TJ, Ranade S, et al (2010) Piezo1 and Piezo2 are essential components of distinct mechanically activated cation channels. Science 330: 55–60 doi:10.1126/science.1193270. 20813920 10.1126/science.1193270 PMC3062430

[45] Kim SE, Coste B, Chadha A, Cook B, Patapoutian A (2012) The role of Drosophila Piezo in mechanical nociception. Nature 483: 209–212 doi:10.1038/nature10801. 22343891 10.1038/nature10801 PMC3297676

[46] Kang D, Choe C, Cavanaugh E, Kim D (2007) Properties of single two-pore domain TREK-2 channels expressed in mammalian cells. J Physiol (Lond) 583: 57–69 doi:10.1113/jphysiol.2007.136150. 17540699 10.1113/jphysiol.2007.136150 PMC2277227

[47] Brohawn SG, del Mármol J, MacKinnon R (2012) Crystal structure of the human K2P TRAAK, a lipid- and mechano-sensitive K+ ion channel. Science 335: 436–441 doi:10.1126/science.1213808. 22282805 10.1126/science.1213808 PMC3329120

[48] Sheetz MP, Singer SJ (1974) Biological membranes as bilayer couples. A molecular mechanism of drug-erythrocyte interactions. Proc Natl Acad Sci USA 71: 4457–4461 doi:10.1016/j.jtbi.2012.01.039. 4530994 10.1073/pnas.71.11.4457 PMC433905

[49] Hill K, Schaefer M (2007) TRPA1 is differentially modulated by the amphipathic molecules trinitrophenol and chlorpromazine. J Biol Chem 282: 7145–7153 doi:10.1074/jbc.M609600200. 17218316 10.1074/jbc.M609600200

[50] Haswell ES, Phillips R, Rees DC (2011) Mechanosensitive channels: what can they do and how do they do it? Structure 19: 1356–1369 doi:10.1016/j.str.2011.09.005. 22000509 10.1016/j.str.2011.09.005 PMC3203646

[51] Kung C (2005) A possible unifying principle for mechanosensation. Nature 436: 647–654 doi:10.1038/nature03896. 16079835 10.1038/nature03896

[52] Delmas P, Hao J, Rodat-Despoix L (2011) Molecular mechanisms of mechanotransduction in mammalian sensory neurons. Nat Rev Neurosci 12: 139–153 doi:10.1038/nrn2993. 21304548 10.1038/nrn2993

[53] Wood AJ, Campagna JA, Miller KW, Forman SA (2003) Mechanisms of actions of inhaled anesthetics. New England Journal of Medicine 348: 2110–2124. 12761368 10.1056/NEJMra021261

[54] Botelho AV, Huber T, Sakmar TP, Brown MF (2006) Curvature and hydrophobic forces drive oligomerization and modulate activity of rhodopsin in membranes. Biophys J 91: 4464–4477 doi:10.1529/biophysj.106.082776. 17012328 10.1529/biophysj.106.082776 PMC1779922

[55] Baciu M, Holmes MC, Leaver MS (2007) Morphological transitions in model membrane systems by the addition of anesthetics. J Phys Chem B 111: 909–917 doi:10.1021/jp066595n. 17249835 10.1021/jp066595n

[56] Braun G, Nemcsics B, Enyedi P, Czirják G (2011) TRESK background K(+) channel is inhibited by PAR-1/MARK microtubule affinity-regulating kinases in Xenopus oocytes. PLoS ONE 6: e28119 doi:10.1371/journal.pone.0028119. 22145024 10.1371/journal.pone.0028119 PMC3228728

[57] Barnes AP, Polleux F (2009) Establishment of axon-dendrite polarity in developing neurons. Annual Review of Neuroscience 32: 347–381 doi:10.1146/annurev.neuro.31.060407.125536. 10.1146/annurev.neuro.31.060407.125536 PMC3170863 19400726

[58] Shelly M, Poo M-M (2011) Role of LKB1-SAD/MARK pathway in neuronal polarization. Dev Neurobiol 71: 508–527 doi:10.1002/dneu.20884. 21416623 10.1002/dneu.20884

[59] Klein AH, Sawyer CM, Zanotto KL, Ivanov MA, Cheung S, et al (2011) A tingling sanshool derivative excites primary sensory neurons and elicits nocifensive behavior in rats. Journal of Neurophysiology 105: 1701–1710 doi:10.1152/jn.00922.2010. 21273322 10.1152/jn.00922.2010 PMC3075278

[60] Albin KC, Simons CT (2010) Psychophysical evaluation of a sanshool derivative (alkylamide) and the elucidation of mechanisms subserving tingle. PLoS ONE 5: e9520 doi:10.1371/journal.pone.0009520. 20209090 10.1371/journal.pone.0009520 PMC2831077

[61] Bryant BP, Mezine I (1999) Alkylamides that produce tingling paresthesia activate tactile and thermal trigeminal neurons. Brain Research 842: 452–460. 10526142 10.1016/s0006-8993(99)01878-8

[62] Basbaum AI, Bautista DM, Scherrer G, Julius D (2009) Cellular and molecular mechanisms of pain. Cell 139: 267–284 doi:10.1016/j.cell.2009.09.028. 19837031 10.1016/j.cell.2009.09.028 PMC2852643

[63] Sung B, Wang S, Zhou B, Lim G, Yang L, et al (2007) Altered spinal arachidonic acid turnover after peripheral nerve injury regulates regional glutamate concentration and neuropathic pain behaviors in rats. Pain 131: 121–131 doi:10.1016/j.pain.2006.12.020. 17267128 10.1016/j.pain.2006.12.020 PMC2478515

[64] Schmidt R, Schmelz M, Torebjörk HE, Handwerker HO (2000) Mechano-insensitive nociceptors encode pain evoked by tonic pressure to human skin. Neuroscience 98: 793–800. 10891622 10.1016/s0306-4522(00)00189-5

[65] Schmidt R, Schmelz M, Forster C, Ringkamp M, Torebjörk E, et al (1995) Novel classes of responsive and unresponsive C nociceptors in human skin. J Neurosci 15: 333–341. 7823139 10.1523/JNEUROSCI.15-01-00333.1995 PMC6578337

[66] Andres-Enguix I, Shang L, Stansfeld PJ, Morahan JM, Sansom MSP, et al (2012) Functional analysis of missense variants in the TRESK (KCNK18) K channel. Sci Rep 2: 237 doi:10.1038/srep00237. 22355750 10.1038/srep00237 PMC3266952

[67] Zhang X, Levy D, Noseda R, Kainz V, Jakubowski M, et al (2010) Activation of meningeal nociceptors by cortical spreading depression: implications for migraine with aura. Journal of Neuroscience 30: 8807–8814 doi:10.1523/JNEUROSCI.0511-10.2010. 20592202 10.1523/JNEUROSCI.0511-10.2010 PMC2907647

